# Quercetin-Coating Promotes Osteogenic Differentiation, Osseointegration and Anti-Inflammatory Properties of Nano-Topographic Modificated 3D-Printed Ti6Al4V Implant

**DOI:** 10.3389/fbioe.2022.933135

**Published:** 2022-06-08

**Authors:** Nian Liu, Hui Wang, Zeyu Fu, Chuxi Zhang, Wenyu Hui, Jinyang Wu, Yong Zhang, Shilei Zhang

**Affiliations:** ^1^ Department of Oral and Cranio-Maxillofacial Surgery, Shanghai Ninth People’s Hospital, College of Stomatology, Shanghai Jiao Tong University School of Medicine, National Clinical Research Center for Oral Diseases, Shanghai Key Laboratory of Stomatology and Shanghai Research Institute of Stomatology, Shanghai, China; ^2^ School of Materials and Chemistry, University of Shanghai for Science and Technology, Shanghai, China

**Keywords:** quercetin, three-dimensional printing, titanium alloy, micro-nano-topography, rapid osseointegration, macrophage polarization, anti-inflammation

## Abstract

The capabilities of osseointegration and anti-inflammatory properties are of equal significance to the bio-inert titanium implant surface. Quercetin has proved its capacities of activating anti-inflammation through macrophage modulation and promoting osteogenic differentiation. Herein, we fabricated quercetin-coating on nano-topographic modificated 3D-printed Ti6Al4V implant surface. Subsequently the biological cells responses *in vitro*, anti-inflammatory and osseointegration performance *in vivo* were evaluated. *In vitro* studies indicated that quercetin-coating can enhance the adhesion and osteogenic differentiation of rBMSCs, while modulating the polarization of macrophages from M1 to M2 phase and improving the anti-inflammatory and vascular gene expression. Moreover, quercetin-loaded implants reduced the level of peri-implant inflammation and ameliorated new bone formation and rapid osseoinegration *in vivo*. Quercetin-coating might provide a feasible and favorable scheme for endowing 3D-printed titanium alloy implant surface with enhanced the rapid osseointegration and anti-inflammatory properties.

## 1 Introduction

Restoring large bone defects caused by tumor, trauma and osteoporosis is undoubtedly a great challenge, especially in load-bearing areas such as jaws and limbs (Hassan et al., 2019). The clinical use of autogenous bone grafts, as the current gold standard treatment, is limited due to the lack of donor site availability. Three-dimensional (3D) printed bone substitutes have been applied to produce almost all kinds of biomaterials in clinical practice (Bose et al., 2018), exhibiting multiple advantages like design flexibility and higher efficiency.

Titanium and its alloys are widely used in clinic because of their superior in mechanical properties and biocompatibility. Moreover, the extensibility of metal can realize the personalized and precise restoration through 3D printing. Yet the biological inertia of titanium alloys leads to unsatisfying long-term implant survival, for the nonbiological Ti implants may induce a soft foreign body response that results in fibrous tissue formation (Goodman et al., 2013), infections and bone resorption in the implanted area (Civantos et al., 2017), thus hindering its potential clinical application.

The surface properties of implant materials, such as surface morphology and chemical composition, can directly impact the biological effects of cell adhesion, proliferation, differentiation, and ultimately affect the quality of osseointegration between implant and host bone (Chen et al., 2016). Current major strategies of titanium surface modification include physical modification, chemical modification and biochemical modification. With the advance of biochemical surface modification, bioactive agents such as protein, peptide, growth factor and drugs have been tentatively applied to implant surfaces, endowing the materials with multiple functions such as osteoinduction, osteoconduction and anti-inflammation.

Host’s inflammatory response to the implantation is inevitable, which is an essential process of tissue regeneration. Implant osseointegration originates from the inflammatory driving process on and near the implant surface (Zhao et al., 2012). Before osteogenesis and angiogenesis, the initial inflammatory response of immune cells [macrophages (m Φ)/monocytes] to the surface of the material determines the fate of the implant. Macrophages are plastic and dynamic that can polarize to classically activated inflammatory phenotype (M1) or alternatively activated inflammatory macrophages (M2) when stimulated by different signals (Kang et al., 2017). Characteristic M1 pro-inflammatory profile exerts a strong cytotoxic activity through production of nitric reactive species (inducible nitric oxide synthase, iNOS), apart from a Th1 pro-inflammatory response [interleukin-1β (IL-1β), IL-6] (Van den Bossche et al., 2017). Macrophages with this phenotype are beneficial for pathogens/tumour elimination but detrimental for the wound healing process (Zhang and Mosser, 2008). On the other hand, M2 anti-inflammatory profile, with mannose receptor (CD206) as typical surface markers, contributes to inflammation resolution and wound healing by producing anti-inflammatory cytokines such as IL-10 and angiogenesis mediators such as transforming growth factor-β (TGF-β) and vascular endothelial growth factor (VEGF) (Funes et al., 2018). M1 and M2 macrophages can transform into each other under external stimulation, and the transformation from M1 to M2 is the turning point from inflammation stage to repair stage (Landén et al., 2016). This functional plasticity of macrophages is the premise that the implant surface can play an immunomodulatory role. The physical and chemical properties of implant surface can affect the polarization of macrophages, and then affect the direction, degree and scope of the inflammatory process. Therefore, the design of implant materials should actively regulate the process of the inflammatory reaction rather than avoiding it, so as to make it turn to the direction conductive to tissue regeneration.

Recent reports threw light on the field that nano-structured surface can regulate the function of inflammatory response related cells, especially the function of macrophages by modulating the polarization between M1 and M2 phenotypes of macrophages and the secretion of cytokines (Vishwakarma et al., 2016). In addition to the change of implant surface structure, the introduction of various bioactive molecules (e.g., functional elements, growth/differentiation cytokines and small molecule drugs) loaded on the biomaterial surfaces can harness macrophage polarization to generate an osteogenic immune microenvironment, so as to regulate the direction, scope and degree of inflammation, which is ultimately beneficial to bone tissue regeneration (Chen et al., 2017; Dong et al., 2017). Surface modification with dual functions of enhancing osteogenesis and regulating macrophage polarization may be a promising solution to the bio-inertia of titanium alloy implants.

Quercetin is a flavonoid monomer compound of small polyphenolic molecules which widely exists in natural plants. It has many pharmacological effects such as anti-inflammation, anti-oxidation, anti-tumor, hypoglycemic and hypolipidemic (Russo et al., 2012). Recent reports illustrated that introducing quercetin onto nano-octahedral ceria could modulate the phenotypic switch of macrophages by not only inhibition of M1 polarization but also promotion of M2 polarization in periodontal disease (Wang Y et al., 2021). Meanwhile, numerous reports have confirmed its impacts on osteogenesis. Quercetin stimulated ALP activity of mesenchymal stem cells (MSCs) in a dose-dependent manner and up-regulated the expressions of ontogenetic marker proteins BGP and COL-1 besides the stimulation of MAPK/ERK signal pathway (Li et al., 2015). Quercetin could also promote OVX rBMSCs proliferation, osteogenic differentiation and angiogenic factor expression while rebuilding the balance of the RANKL/OPG system in a dose-dependent manner (Zhou et al., 2017). To sum up, quercetin might constitute an appropriate candidate as the loaded drug of titanium alloy implants to regulate macrophages polarization and enhance the osteogenesis at the same time.

However, in the deficiency of active functional groups on titanium surface, how to realize the effective loading of quercetin is an urgent problem to be resolved. Our previous research has successfully fabricated hierarchical micro/nano-topography on the Ti6Al4V implant surface through the combination of 3D-printing, alkali-heat treatment and subsequent hydrothermal treatment (Wang H et al., 2021). The graded micro/nano-topography, deposited with anatase phase of titanium dioxide (TiO_2_) on the surface, possessed a high specific surface area to increase the adsorption of specific proteins that leading to better biocompatibility. Since quercetin has strong power to chelate metal cations (Sun et al., 2008), the drug was observed to be absorbed on TiO_2_ in monomeric form by bidentate chelating the Ti atom in TiO_2_ through two dissociated hydroxy functions at the catechol ring B (Zdyb and Krawczyk, 2016). 3D-printed Ti6Al4V implant with micro/nano-topography could also provide more binding sites for quercetin, so as to improve the drug-loading efficiency. Therefore, micro-nano hybrid 3D-printed titanium surface may be an ideal delivery carrier of quercetin.

In this study, we constructed the nano-topographic surface on micro-scaled 3D-printed Ti6Al4V implants on the basis of our prior research, then introduced quercetin-coating on the implant surface. The regulation on the biological behavior of macrophages and rBMSCs stimulated by quercetin-loading was evaluated, along with the observation of anti-inflammation and osseointegration performance in animal models.

## 2 Materials and Methods

### 2.1 Materials Preparation

In this study, 3D-printed Ti6Al4V (Ti) samples were prepared as two shapes: square disks (10 mm in side length, 2 mm in thickness) and rod-like implants (2 mm in diameter, 3.5 mm in length), both were fabricated from 20 to 50 μm Ti6Al4V alloy powders as described in previous study (Zhang et al., 2019).

The samples were thoroughly ultrasonic cleaned in acetone, ethanol and distilled water to remove the adhered particles and then placed in polytetrafluoroethylene-lined metal reaction kettle with NaOH solution (5 mol/L) at 80°C for 6 h and next in deionized water at 200°C for 4 h to obtain the nanostructured topography, namely the nano-3D group (Wang et al., 2018).

Nano-3D samples were cleaned with deionized water, steam autoclaved and dried as described before *in vitro* or *in vivo* studies. Half of the nano-3D disks/implants were soaked into quercetin solution for drug-loading. Each piece of nano-3D samples was immersed in quercetin ethanol solution (0.05 mg/ml, 10 ml) in a 15 ml centrifuge tube. The samples were ultrasonic treated for 5 min and then placed at 4°C for 1 h. After gentle rinse with deionized water to remove the non-adsorbed quercetin, the nano-3D + quercetin samples were dried at room temperature and UV light sterilized for standby.

### 2.2 Surface Characterization of Materials

The surface topography of both groups was observed via scanning electron microscope (GeminiSEM 300, ZEISS, Germany). Raman spectroscope (RW 2000, Renishaw, England) was utilized to verify whether the quercetin was coated on the sample surface. The release of quercetin from the samples was determined by using a UV-vis spectrophotometer (IMPLEN, Germany). Quercetin-loaded nano-3D disks were soaked in phosphate buffer saline (PBS, Hyclone, United States) and shaken with 100 rpm at 37°C. The PBS was collected at 1, 4, 8, 12, 18, 24, 36 h and 2, 3, 4, 5, 6 days respectively, and the concentration of quercetin released was observed at 254 nm wavelength. The data were presented as the percentage of cumulative release in total: Cumulative amount of release (%) = 100 × M_*t*_/M (M_*t*_ for the amount of quercetin released at time *t*; M for the total amount of quercetin). The surface wettability was observed by Optical Contact Angle and Interface Tension Meter SL200KS (SOLON TECK, China).

### 2.3 *In Vitro* Study

#### 2.3.1 Cell Culturing

We employed macrophages (RAW 264.7 from Shanghai cell bank of Chinese Academy of Sciences) and rat BMSCs (rBMSCs) in the present study. The latter was isolated from two-week-old male Sprague Dawley rats (from Shanghai Bikai Animal Laboratory, China). Rats were sacrificed through cervical dislocation after general anesthesia, then peripheral soft tissue of dissected femora and tibiae was removed. The bone was resected at both sides of the metaphysis, then bone marrow contents were flushed into 10 cm cell culture dish containing alpha-modified Eagle’s medium (α-MEM, Hyclone, United States) supplemented with 10% fetal bovine serum (FBS, Hyclone, United States) and 1% penicillin/streptomycin solution (Gibco, United States). The rBMSCs were incubated at 37°C in 5% CO_2_ and the media was changed each 2 days, and third passages of rBMSCs were used in the following experiments**.**

#### 2.3.2 Cell Adhesion and Proliferation

To assess the morphology of cells adhered on the samples, cells (RAW264.7: 1 × 10^5^/ml, rBMSCs: 1 × 10^4^/ml) were seeded onto each sample in a 24-well plate and cultured for 24 h respectively. Then samples were rinsed with PBS and fixed in 2.5% glutaraldehyde at 4°C overnight. After dehydration in graded ethanol series sequentially, samples were freezing dried and sputter coated with gold before SEM scanning (S-4800, Hitachi, Japan).

Cell proliferation was evaluated through Cell Counting Kit-8 (CCK-8, Beyotime, China) after culturing rBMSCs for 1, 4, 7 days and RAW 264.7 for 1, 3, 5 days respectively. Briefly, cells were seeded on samples (RAW264.7: 1 × 10^5^/well, rBMSCs: 1 × 10^4^/well) in 24-well plates and the media was changed every 2 days. At each time point, every sample was transtrred into a new well and incubated in CCK-8 working solution for 2 h. The absorbance was measured at 450 nm wavelength using a microplate reader (Spectra Max M5, Molecular Devices, United States).

#### 2.3.3 Quercetin-Coated Surface Modulating RAW 264.7 Polarization

After incubation on samples for 24 h, the culture medium of macrophages was collected and centrifuged. ELISA kits (Boster, China) were used to examine the concentrations of IL-1β, VEGF-α and TGF-β in the supernatants according the manufacturer’s instructions.

The gene expression level was determined by quantitative real-time polymerase chain reaction (qRT-PCR) assay so as to evaluate the related genes expression. The RAW 264.7 were seeded with a density of 4×10^6^/well on the sample surfaces in 6-well plates. Total RNA was extracted and separated by RNAfast200 RNA Isolation Kits (Feijie, China) after 3 days, then reversely transcribed into cDNA through a Prime-Script RT reagent kit (Takara, Japan). The cDNA samples were 1:10 diluted in RNase-free water and stored at −20°C until the PCR reaction was performed. Primers used in the present study were synthesized commercially (Sangon, China), and are set out in Table 1. The real-time PCR procedure was performed with SYBR green PCR reaction mix (Takara, Japan) in Light Cycler^®^ 96 Real-Time PCR System (Roche, Switzerland).

**TABLE 1:** Primer squences for real-time PCR.

Gene	Primers (F = forward; R = reverse)
VEGF-α	F: 5′-GTC​CCA​TGA​AGT​GAT​CAA​GTT​C-3′
	R: 5′-TCT​GCA​TGG​TGA​TGT​TGC​TCT​CTG-3′
TGF-β	F: 5′-CAG​TAC​AGC​AAG​GTC​CTT​GC-3′
	R: 5′-ACG​TAG​TAG​ACG​ATG​GGC​AG-3′
IL-10	F: 5′-GCT​CTT​ACT​GAC​TGG​CAT​GAG-3′
	R: 5′-CGC​AGC​TCT​AGG​AGC​ATG​TG-3
CD206	F: 5′-AGA​CGA​AAT​CCC​TGC​TAC​TG-3′
	R: 5′-CAC​CCA​TTC​GAA​GGC​ATT​C-3′
INOS	F: 5′-GTT​CTC​AGC​CCA​ACA​ATA​CAA​GA-3′
	R: 5′-GTG​GAC​GGG​TCG​ATG​TCA​C-3
IL-1β	F: 5′-GCA​ACT​GTT​CCT​GAA​CTC​AAC​T-3′
	R: 5′-GCA​ACT​GTT​CCT​GAA​CTC​AAC​T-3′
GAPDH	F: 5′-AGG​TCG​GTG​TGA​ACG​GAT​TTG-3′
	R: 5′-TGT​AGA​CCA​TGT​AGT​TGA​GGT​CA-3

#### 2.3.4 Quercetin-Coated Surface Promoting rBMSCs Osteogenic Differentiation

Alkaline phosphatase (ALP) activity and staining assay of rBMSCs cultured on the discs were measured at 4 and 7 days. Cells were seeded at a density of 4 × 10^4^/well and at each time point, the samples were rinsed with PBS. For ALP staining, cells were fixed with 4% paraformaldehyde (PFA) and stained with ALP Color Development Staining Kit (Beyotime, China). After 12 h, stained materials were observed through optical microscope (Olympus, Japan). While for ALP activity assay, cells were lysed in 0.1% Triton ×100 buffer (Beyotime, China). After centrifugation, the supernatant was used to detect the ALP activity *via* ALP Assay Kit (Jiancheng, China), and the total protein concentration was determined with BCA Protein assay kit (Beyotime, China) according to the manufacturer’s instruction. Finally, the ALP activity was calculated and normalized to the total protein level (U/g protein).

Besides, qRT-PCR assay was employed to investigate the related genes expression at 4 and 7 days so as to evaluate the effect of quercetin-coating on osteogenic differentiation, primers in this part are describedd in Table 2.

**TABLE 2:** Primer squences for real-time PCR.

Gene	Primers (F = forward; R = reverse)
OCN	F: 5′-GCC​CTG​ACT​GCA​TTC​TGC​CTC​T-3′
	R: 5′-TCA​CCA​CCT​TAC​TGC​CCT​CCT​G-3
COL-I	F: 5′-GCC​TCC​CAG​AAC​ATC​ACC​TA-3′
	R: 5′-GCA​GGG​ACT​TCT​TGA​GGT​TG-3′
ALP	F: 5′-TAT​GTC​TGG​AAC​CGC​ACT​GAA​C-3′
	R: 5′-CAC​TAG​CAA​GAA​GAA​GCC​TTT​GG-3′
OPN	F: 5′-CCA​AGC​GTG​GAA​ACA​CAC​AGC​C-3′
	R: 5′-GGC​TTT​GGA​ACT​CGC​CTG​ACT​G-3
β-actin	F: 5′-GTA​AAG​ACC​TCT​ATG​CCA​ACA-3′
	R: 5′-GGA​CTC​ATC​GTA​CTC​CTG​CT-3′

### 2.4 *In Vivo* Study

#### 2.4.1 Surgical Procedures

SD rats weighted approximately 250 g were used in the present study. General anesthesia was conducted by intraperitoneal injection with pentobarbital sodium (30 mg/kg, Beyotime, China) and then the surgical area was shaved and washed with povidone iodine. A 1 cm-long incision was made through the skin, muscle and periosteum at the lateral side of femoral condyles to expose the implantation position. Subsequently, a hole of 2 mm in diameter and 3 mm in depth was prepared with a bur and the drilling procedure was accompanied with constant irrigation of sterile saline. Implants of nano-3D and nano-3D + quercetin were inserted into the left or right femoral condyle randomly and finally tissues were sutured in layers.

Animals were euthanized after 2 or 4 weeks of healing (n = 6 for each time point) and then the femoral condyles were resected and fixed in 4% PFA for further analysis.

#### 2.4.2 Micro-Computed Tomography (Micro-CT Assay)

In order to evaluate the peri-implant new bone volumes *in vivo*, the harvested samples were scanned through micro-CT scanning system (Quantum GX, United States). The scanning parameters were set at 90kV, 88 μA and 14 min, then the images were 3D-reconstructed in the voxel size of 25 μm, so as to calculate the ratio of bone volume to total volume (BV/TV) with the attached analysis software.

#### 2.4.3 Histological and Histomorphometric Analysis

Hematoxylin and eosin (H&E) and immunofluorescence (IF) staining were performed on the peri-implant tissues harvested at 2 weeks. After decalcification for 1 month, the implants can be softly screwed out of the samples with tweezers, then the remaining tissues were embedded in paraffin and sectioned into 5 μm slices. The sections were stained with H&E to validate the inflammatory level, and the IF staining was employed to evaluate the macrophage phenotypes infiltrating the peri-implant tissues. The staining procedures were conducted according to the manufacturer’s instruction with the antibodies (Affinity, United States).

The samples harvested at 4 weeks were dehydrated in graded ethanol series from 70% to 100% sequentially and embedded in methyl methacrylate (MMA) for undecalcified sectioning. The polymerized samples were longitudinally sectioned and polished with a Diamond Circular Saw Microtome and Micro Grinding System (Exakt 300, Germany). The sections were stained with Van Gieson’s (VG) staining kit (Yuanye, China) and visualized under a light microscope (Olympus, Japan) for histological observation. For histomorphometric measurements, pictures captured by the digital camera attached to the microscope were analyzed, and the bone-to-implant contact (BIC) percentage was calculated *via* ImageJ.

### 2.5 Statistical Analysis

All quantitative data were performed as mean ± standard deviation (SD) and were statistically analyzed by *t*-test through GraphPad Prism 8.0 software. The difference was considered significant when *p* value was less than 0.05.

## 3 Results and Discussion

### 3.1 Surface Characterization

**FIGURE 1:**
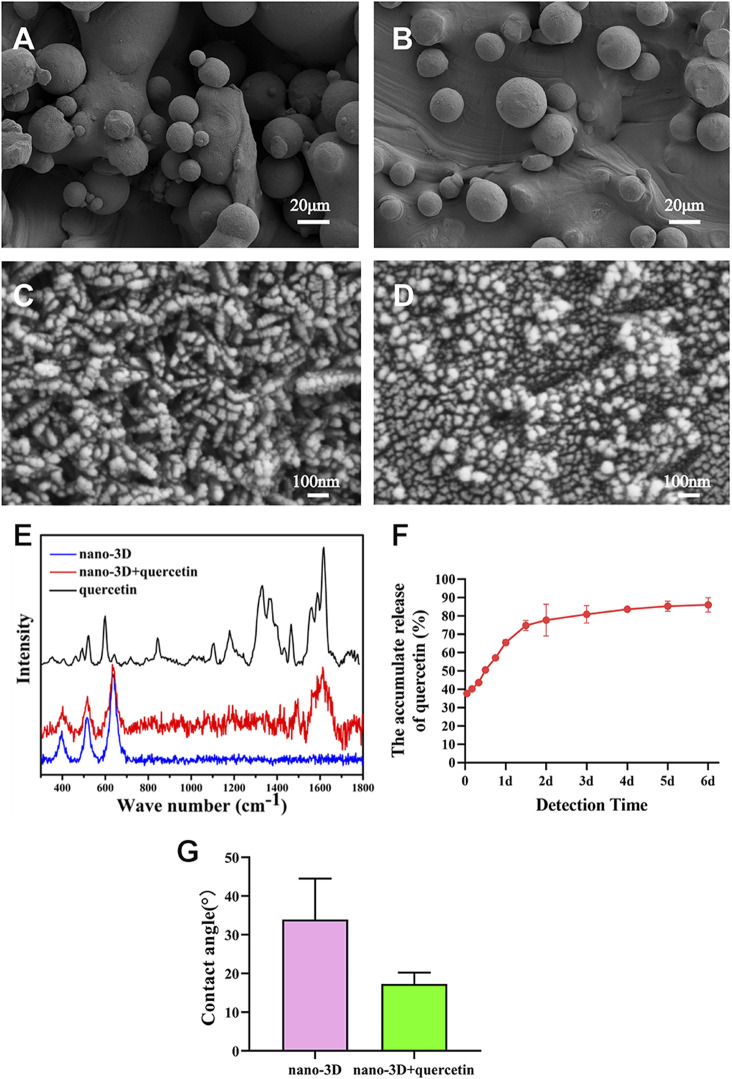
SEM images of nano-3D **(A,C)** and nano-3D + quercetin **(B,D)** at low **(A,B)** and high magnifications **(C,D)**; Raman spectra analysis **(E)**, accumulative release percentage of quercetin loaded on the nano-3D samples at different times **(F)** and water contact angle **(G)** of nano-3D/nano-3D + quercetin sample surfaces (^*^ *p* < 0.05).

As presented in Figures 1A–D, there was no significant difference in surface morphology between nano-3D and nano-3D + quercetin. This may be due to the fact that quercetin is a small molecular substance that cannot be observed on SEM. The Raman spectrum (Figure 1E) showed typical quercetin peak appeared on the surface of nano-3D + quercetin at about 1606 cm^−1^, which indicated that quercetin had been successfully loaded on the surface of titanium scaffolds. The results of accumulative quercetin release (Figure 1F) showed significant quercetin release at 37.70 ± 0.39% in the first hour. Afterwards the quercetin release displayed a linear trend of steep increase to 74.75 ± 2.78% at 36 h, then gently increased until reaching 86.01 ± 3.91% at 6 days. These results implied that the nano-3D titanium alloy disks as a quercetin delivery carrier could provide effective and stable drug release, and the release amount could quickly reach a high level at the first 2 days. Water contact angle analysis showed that the surface water contact angle of nano-3D + quercetin sample was lower than that of nano-3D group, but the difference was not statistically significant (Figure 1G). This may indicate that quercetin loading has little effect on the surface hydrophilicity of nano-modified 3D-printing implants.

### 3.2 Cell Adhesion and Proliferation

**FIGURE 2:**
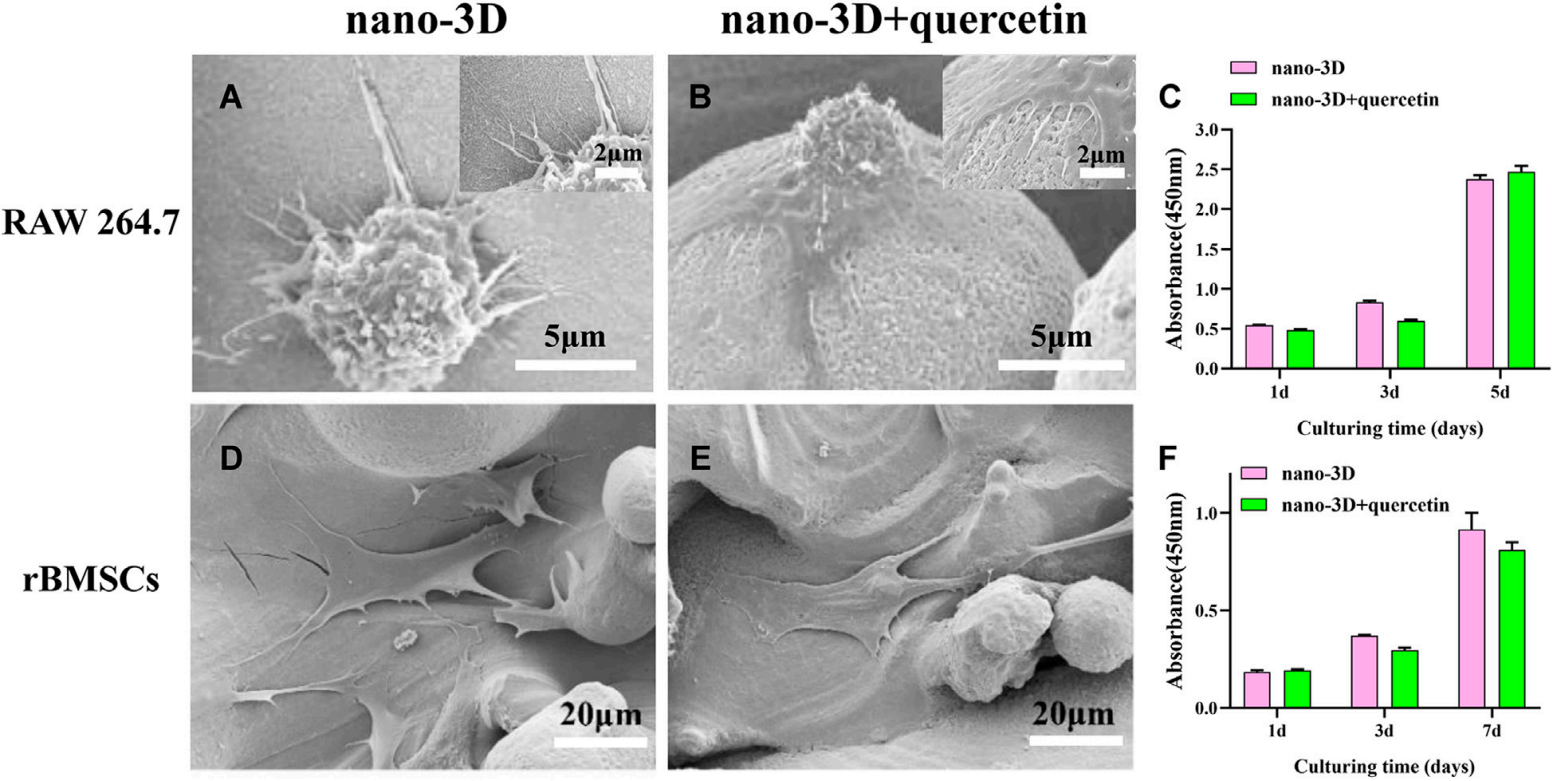
SEM images of adherent RAW 264.7 **(A,B)** and rBMSCs **(D–E)** cultured on nano-3D **(A,D)** and nano-3D + quercetin **(B,E)**; CCK-8 analysis of RAW 264.7 **(C)** and rBMSCs **(F)** cultured on nano-3D and nano-3D + quercetin samples (^*^ *p* < 0.05).

The SEM results of 24 h adhesion of RAW 264.7 (Figures 2A,B), the macrophages on both groups exhibited almost spherical shape, yet macrophages on the nano-3D + quercetin samples had more pseudopods, indicating more sufficient adhesion and spreading. As polarized M1 macrophages displayed round-like shape without any spreading in general, while M2 macrophages exhibiting spindle-shaped and better spreading morphology, the results might suggest that macrophages on the quercetin-loaded samples were more likely to polarize into M2 phenotype at the first day. Similar trends were also detected in the results of rBMSCs (Figures 2D,E), for that the cells on the surface of nano-3D + quercetin group were better adhered and spread with more plate-like and filiform pseudopods. Good adhesion plays an important role in cell proliferation and differentiation, however, CCK-8 analysis results (Figures 2C,F) showed no significant difference between the two groups, neither in rBMSCs nor in macrophages, indicating good cytocompatibilities of the quercetin-loading and quercetin at this concentration does not promote cell proliferation.

### 3.3 Quercetin-Coated Surface Modulating RAW 264.7 Polarization

**FIGURE 3:**
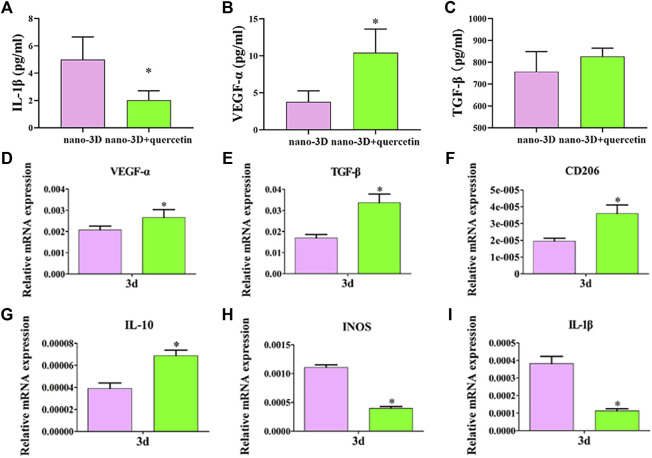
Elisa results of IL-1β, VEGF-α and TGF-β at 24 h respectively **(A–C)** and relative genes expression of RAW 264.7 **(D–I)** on nano-3D and nano-3D + quercetin samples at 3 days (^*^ *p* < 0.05).

To further verify the representative cytokines secreted by macrophages in M1/M2 phenotype, ELISA was implemented to determine the concentrations of IL-1β, VEGF-α and TGF-β. The results are shown in Figures 3A–C. The expression levels of IL-1β, the typical inflammatory cytokine mainly secreted by M1 macrophages, was significantly lower on quercetin-coated samples (Figure 3A). In contrast, macrophages on nano-3D + quercetin secreted the greater amounts of the anti-inflammatory cytokine VEGF-α largely produced by M2 macrophages (Figure 3B), yet no significant difference shown in TGF-β production between the two groups (Figure 3C).

Highly consistent with the ELISA results, as is presented in Figures 3D–I, the nano-3D + quercetin group was more conductive to the expression of anti-inflammatory phenotype (M2) related genes, such as VEGF-α, TGF-β, IL-10 and CD206, while reducing the expression of activated inflammatory macrophage (M1) related genes, such as iNOS and IL-1β, indicating that quercetin-coating could regulate the polarization of macrophages to M2 macrophages. M2 phenotype macrophages secrete a variety of immunoregulatory factors and chemokines to recruit and integrate fibroblasts, bone marrow mesenchymal cells, endothelial cells and other repair cells to the wound, thereby maintaining the tissue homeostasis and promoting the inflammatory response to enter the stage of tissue regeneration as soon as possible.

### 3.4 Quercetin-Coated Surface Promoting rBMSCs Osteogenic Differentiation

**FIGURE 4:**
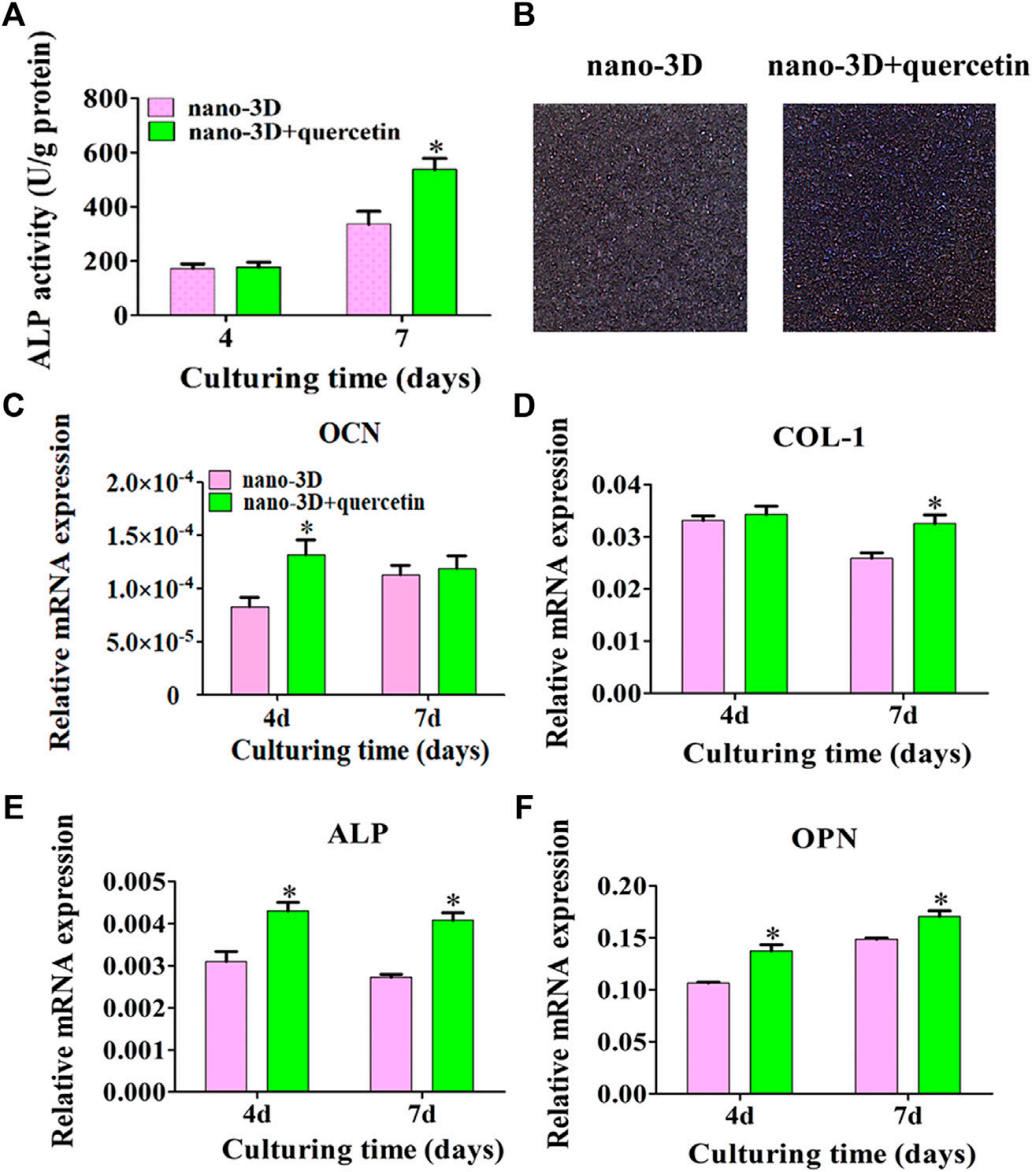
ALP activity at 4/7 days **(A)** and staining at 7 days **(B)** of rBMSCs on nano-3D and nano-3D + quercetin sheets; Expression levels of OCN **(C)**, COL- I **(D)**, ALP **(E)** and OPN **(F)** in rBMSCs cultured on nano-3D and nano-3D + quercetin sheets at 4/7 days (^*^ *p* < 0.05).

At 4 days, there was no significant difference in ALP activity between the two groups; at 7 days, the ALP expression on the surface of nano-3D + quercetin group increased significantly than that of nano-3D group (Figure 4A), and the ALP staining results (Figure 4B) confirmed the trend.

As demonstrated in Figures 4C–F, compared with the nano-3D group, the expression of osteogenesis related genes such as OCN, ALP and OPN on the surface of nano-3D + quercetin group increased significantly at 4 days, except that no significant difference shown in the expression of COL- I between the two groups. While at 7 days, the expression of COL-I, ALP and OPN on the surface of nano-3D + quercetin increased significantly, and the difference was statistically significant compared to the other group, besides no significant difference shown in the gene expression of OCN between the two groups at the time.

The results of quantitative detection of ALP expression and qRT-PCR exhibited similar trends that ALP and osteogenic related gene expression were higher in nano-3D + quercetin group, which implied that quercetin-coating was more favorable for osteogenic differentiation of rBMSCs.

### 3.5 Capability of Anti-Inflammation of Quercetin-Coated Nano-3D Implants

**FIGURE 5:**
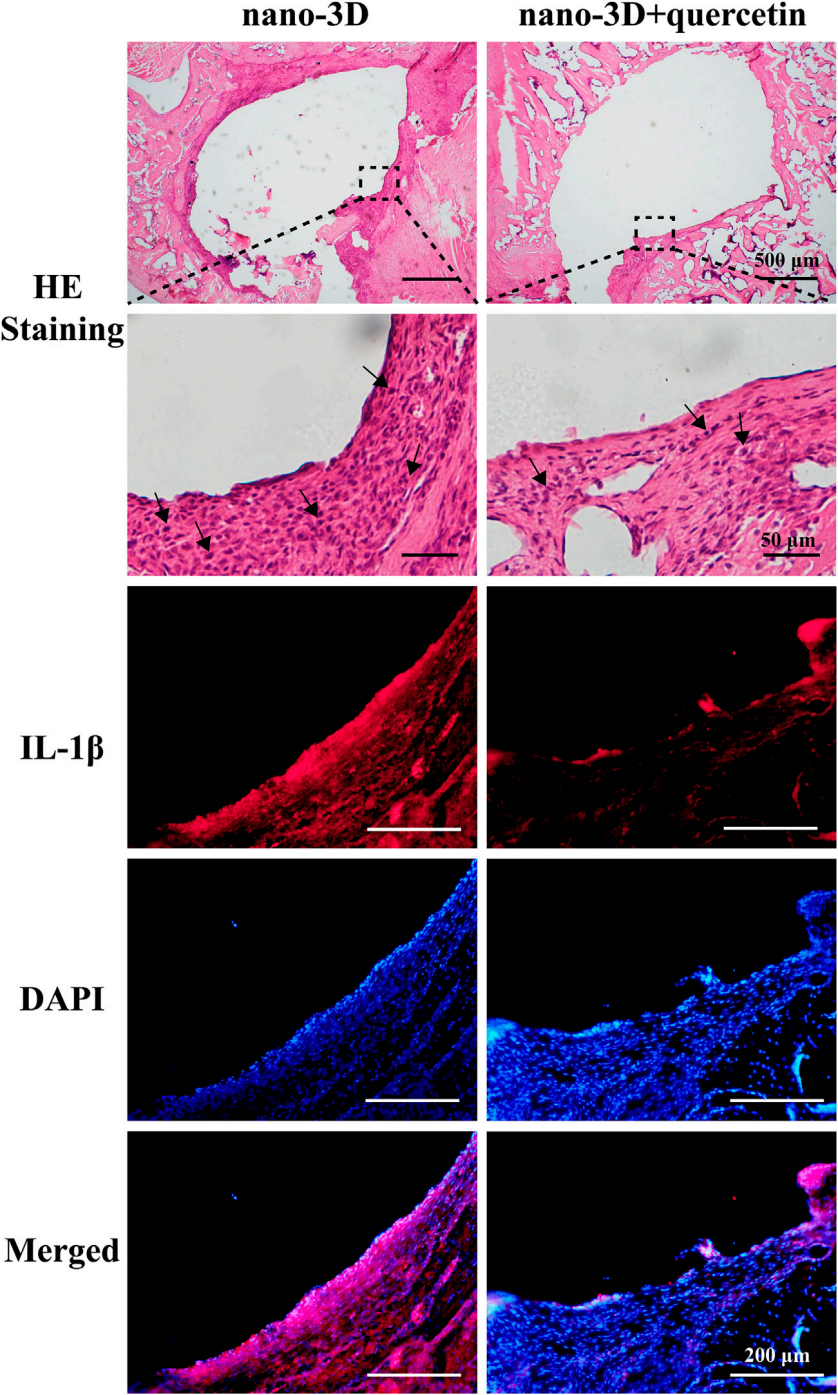
H&E staining of the decalcified peri-implant tissues of nano-3D and nano-3D + quercetin at 2 weeks, black arrows indicate inflammatory cells such as macrophages, neutrophils, monocytes and lymphocytes; immunofluorescent staining results of the decalcified samples: red (IL-1β) and blue (DAPI).

The images of H&E staining in Figure 5 demonstrated the pathological changes in the peri-implant tissues. Compared to the quercetin-loaded group, the nano-3D sections showed more severe inflammatory state with abundant inflammatory cells infiltration, such as monocytes, neutrophils and macrophages. To further investigate the therapeutic effects of quercetin against inflammation, the level of inflammation-associated cytokine in the peri-implant tissues were observed by IF. The representative pro-inflammatory M1 biomarker IL-1β positive cells were notably detected in nano-3D group and significantly decreased in the quercetin-loaded samples, showing the anti-inflammatory effects of quercetin at the inflammatory site.

### 3.6 Capability of Osteogenesis and Osseointegration of Quercetin-Coated Nano-3D Implants

**FIGURE 6:**
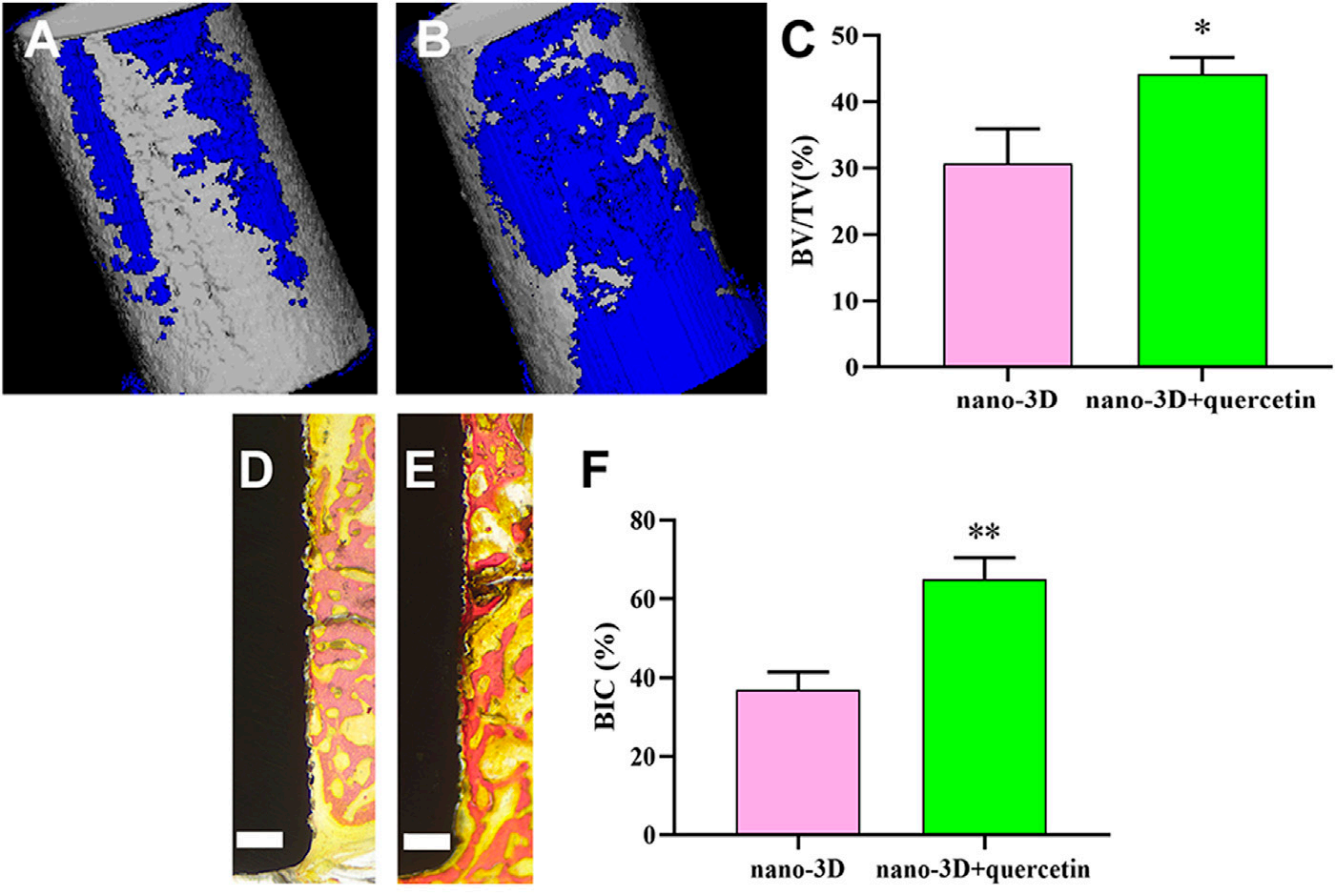
3D-reconstructed images of new bone formation (blue) around the implants (grey) of nano-3D **(A)** and nano-3D + quercetin **(B)** at 4 weeks, quantitative analysis of Micro-CT data: BV/TV ratio **(C)**. Van Gieson staining of undecalcified sections of nano-3D **(D)** and nano-3D + quercetin **(E)** at 4 weeks, scale bar = 300 μm, and the histomorphometric analysis of BIC percentage **(F)**, (^*^ *p* < 0.05, ^**^ *p* < 0.01).

After implantation for 4 weeks, 3D-reconstructed images of nano-3D (Figure 6A) and nano-3D + quercetin (Figure 6B) showed that the volume of new bone formation in the quercetin-coated group was obviously larger than that in the non-coating group. The quantitative results of BV/TV (Figure 6C) also illustrated the difference, which the new bone formation ration of nano-3D + quercetin implants was significantly higher than the other group.

Moreover, the VG staining results of hard tissue slices showed a trend in consistence with the CT analysis, that there was more new bone formed around the surface of quercetin-coated implants (Figure 6E) compared to the nano-3D samples (Figure 6D). The quantitative analysis of new bone area percentages demonstrated that BIC percentage of nano-3D + quercetin group is markedly higher than that of the non-coating group (Figure 6F).

To sum up, histological and histomorphometric results implied that the nano-structural modified 3D-printed Ti6Al4V with quercetin coating could enhance the capacities of osteogenesis and osseointegration around the implants *in vivo*.

Taking the *in vitro* and *in vivo* observations into account, the quercetin-coated nano-topographic modificated 3D-printed Ti6Al4V manifested superiority compared to the control group, which may owe to the capabilities of stimulating osteogenic differentiation and anti-inflammation of quercetin(Angellotti et al., 2020).

## 4 Conclusion

In the present study, we successfully loaded quercetin onto the surface of nano-structural modified 3D-printed Ti6Al4V implants, and then confirmed that quercetin-coating can promote the adhesion of macrophages and modulate the polarization from M1 to M2 phase, thus to improve the anti-inflammatory and vascular gene expression gene expression of the macrophages. Meanwhile, the nano-structural modified 3D-printed Ti6Al4V loaded with quercetin can promote the adhesion and osteogenic differentiation of rBMSCs. Quercetin-loading provided a feasible and favorable scheme for endowing 3D-printed titanium alloy implant surface with enhanced rapid osseointegration and anti-inflammatory properties, and the specific mechanisms of quercetin promoting osteogenesis and anti-inflammation through modulating polarization are worthy of further study.

## Data Availability

The raw data supporting the conclusion of this article will be made available by the authors, without undue reservation.
